# The use of body-worn cameras in pre-hospital resuscitation

**DOI:** 10.29045/14784726.2019.09.4.2.4

**Published:** 2019-09-01

**Authors:** Alistair Dewar, David Lowe, Donald Mcphail, Gareth Clegg

**Affiliations:** Royal Infirmary of Edinburgh; Glasgow Royal Infirmary; University of Edinburgh; Scottish Ambulance Service; Royal Infirmary of Edinburgh

**Keywords:** cardiac arrest, pre-hospital, resuscitation

## Abstract

**Introduction:**

Body-worn cameras (BWCs) are commonplace in many workplaces, but rare in the real-time audit of clinical performance in the pre-hospital setting. There are currently no data supporting the use of BWCs as an acceptable tool in clinical audit. Out-of-hospital cardiac arrest (OHCA) is a good candidate for audit – time critical, high stakes and not well observed. While the use of cameras to record such clinical data is demonstrably useful, it could be perceived by front line ambulance staff as intrusive and have a deleterious impact on clinical care. Investigating these potential barriers is important in ensuring that our effort to enhance the early phase of pre-hospital care through video audit does not have negative unintended consequences.

**Methods:**

Since 2012, the Resuscitation Research Group has used BWCs to provide a unique insight into how care is delivered by paramedics attending OHCAs. Paramedics attending arrests as part of the Resuscitation Rapid Response Unit (3RU) second-tier response wear a BWC, and collect real-time footage of these challenging, emotive clinical encounters. This footage has provided a unique medium for the audit of both individual technical task and team-oriented non-technical skills performance. We present the results of a survey in which paramedics share their views on the use of BWCs within their service.

**Results:**

A convenience sample of 83 questionnaires was collected. In relation to the primary outcome of the study, 81% (n = 53) of paramedics who responded to the statement, ‘the use of BWCs is a positive step for the service’, agreed or remained neutral, while only 19% (n = 12) disagreed.

**Conclusion:**

BWCs, and the supporting infrastructure and feedback processes, are an effective, acceptable and beneficial tool in the audit and analysis of team performance in pre-hospital resuscitation.

## Introduction

Pre-hospital resuscitation is a critical, early link in the ‘chain of survival’ for the management of out-of-hospital cardiac arrest (OHCA). Each OHCA is different, requiring the clinician to exhibit highly attuned technical and non-technical skills in order to balance and prioritise multiple clinical and ergonomic variables effectively (Nolan, Soar, & Eikeland, 2006).

Core to the philosophy of optimising OHCA management is the requirement for high quality data to drive performance improvement. For example, analysis of data from defibrillator downloads provides metrics for several aspects of technical performance such as chest compressions, hands-on time and compression rates and adequacy (Clarke, Lyon, Short, Crookston, & Clegg, 2014; Lyon, Clarke, Milligan, & Clegg, 2012). However, these metrics provide little insight into the ‘non-technical’ elements of OHCA management, including those around leadership, communication, task delegation and team performance (Klein, 1996).

The Resuscitation Rapid Response Unit (3RU) was established in Edinburgh in 2012, as a second-tier response to OHCA providing team-leadership and assisting with critical decision making. Each 3RU paramedic wears a body-worn camera (BWC) (Edesix™ VideoBadge, VB-100/200). The BWC collects point-of-view footage and audio recording from the time of dispatch to an OHCA and continues to record until the case reaches its conclusion, either by halting pre-hospital resuscitation efforts or handover of the patient’s care to the receiving emergency department.

Video is reviewed by the audit team before secure deletion and is not retained as part of the patient’s record. Audit results inform the 3RU training programme, but video is not used for training purposes or individual feedback. All of the front line ambulance staff in the 3RU operational area are aware of the routine use of BWC for audit, and consent for video recording is not sought from staff. The 3RU team will, however, cease recording immediately if requested to do so by another staff member.

As the use of video as an auditing tool in OHCA resuscitation has become established in Edinburgh, we sought to describe the attitudes of ambulance service staff towards the use of BWCs. In particular, we wished to explore the perceived acceptability of their use in the pre-hospital clinical environment.

## Methods

### Questionnaire design

A bespoke questionnaire was created and distributed via SurveyMonkey, designed following the checklist for reporting results of internet e-surveys (CHERRIES) guidance (Eysenbach, 2004).

A series of 15 questions which combined multiple choice, five-point Likert-type scale and free-text questions, was developed and piloted by a group of subject matter experts from the Resuscitation Research Group, University of Edinburgh. Questions were designed to elicit the attitudes of paramedics towards the use of BWCs, the effect of BWCs on clinical practice and performance and feelings towards the use of BWCs as a means of audit and service development in the future.

Participants were made aware that the survey would take around 20 minutes to complete. The survey went through multiple iterations to ensure and optimise clarity of language and a user-friendly appearance. Participants could change their answers at any time before submitting a final completed survey. Responses were collated automatically, and the data then exported and viewed in Microsoft Excel. A full summary of the survey questions is available in Supplementary 1.

### Outcomes

The primary outcome of the survey was the response to the statement, ‘the use of BWCs is a positive step for the service’. Secondary outcomes included perceived impact of the presence of BWCs on the delivery of care and respondents’ views on using BWCs to record their own clinical practice.

### Sample selection

Data were collected from non-3RU paramedics and ambulance technicians working within the same geographical boundaries as the 3RU team. This included ambulance stations where 3RU paramedics were based and therefore crews were likely to have encountered BWCs when attending an OHCA or were aware of their use in clinical practice. Participation was voluntary, and while attempting to gain a representative cross-section of ambulance service staff it was felt that there was no requirement for staff members completing the survey to have previously attended an OHCA where a 3RU paramedic wearing a BWC had been present.

The survey link was distributed by e-mail via ambulance station team leaders, and reminder e-mails sent after 14 and 21 days. No log-in credentials were required to complete the survey, and respondents could opt out of providing demographic data if so desired, remaining fully anonymous and thus promoting a forum for providing honest feedback on sensitive subject material.

## Results

### Sample study characteristics

The survey remained open for a period of 28 days, until there was a 7-day period during which there were no new submissions. This resulted in a convenience sample of 96 questionnaires. Of the 96 submitted surveys, 83 were identified as unique submissions, with duplicate or partial submissions excluded prior to analysis. The ‘reach’ of the survey link was estimated at around 400 paramedics and ambulance technicians, representing a 20% response rate.

Basic demographic information and working patterns are shown in Table 1. Of the 83 unique respondents, 58% were paramedics and 42% were ambulance technicians. Of the respondents, 54% were based primarily at a station within the City of Edinburgh, with 22% of respondents based in stations in East Lothian and Midlothian – the operational areas of these stations all lie within the geographical provision boundaries of the 3RU team. Of the respondents, 7% worked for the Special Operations Response Team at the time of completion. One respondent noted that they did not wish to disclose which station they worked at, so as to remain fully anonymous.

**Table 1.** Demographic data of survey respondents.

	Number
**Age**	
18–24	4 (5%)
25–34	24 (29%)
35–44	24 (29%)
45–54	21 (25%)
55+	9 (11%)
*Did not answer*	*1 (1%)*
**Crew type**	
Paramedic	48 (58%)
Technician	35 (42%)
**Ambulance experience**	
1–5 years	29 (35%)
6–10 years	18 (22%)
11–15 years	13 (15%)
16–20 years	4 (5%)
20+ years	19 (23%)
*Did not answer*	*0*
**Base station by area**	
City of Edinburgh	45 (54%)
West Lothian	11 (13%)
East Lothian	9 (11%)
Midlothian	9 (11%)
SORT	6 (7%)
Borders	2 (3%)
Did not wish to disclose	1 (1%)

Note: SORT = Special Operations Response Team.

### Experience of out-of-hospital cardiac arrest and body-worn cameras

Of the total 83 respondents, 81 provided information on how many OHCAs they had attended in the 12 months immediately preceding the study period. As shown in Table 2, 80% had attended between one and 10 OHCA incidents; 16% had attended more than 10; 3% had not attended any.

**Table 2.** Number of cardiac arrests attended and experience of body-worn cameras.

	Number
**Approximately how many OHCAs have you attended in the last 12 months?**	
Nil	3 (3%)
1–3 incidents	25 (30%)
4–6 incidents	29 (35%)
7–9 incidents	12 (15%)
10+ incidents	13 (16%)
*Did not answer*	*1 (1%)*
**Have you ever been present at an OHCA where a BWC was in use?**	
Yes	83 (100%)
No	0 (0%)
**Have you ever asked the 3RU paramedic to switch their camera off?**	
Yes	11 (13%)
No	56 (67%)
*Did not answer*	*16 (20%)*

Note: 3RU = Resuscitation Rapid Response Unit; BWC = body-worn camera; OHCA = out-of-hospital cardiac arrest.

Of the respondents, 100% had, at some stage, been present at an OHCA where the team leader was wearing a BWC.

There were 67 responses to the question, ‘Have you ever asked the 3RU paramedic to switch their camera off?’. Of these, 11 (13%) answered ‘yes’ (Table 2); 20% of respondents chose not to answer this question, a response rate which is considered further later in the article.

### Body-worn cameras as service provision

**Figure 1:**
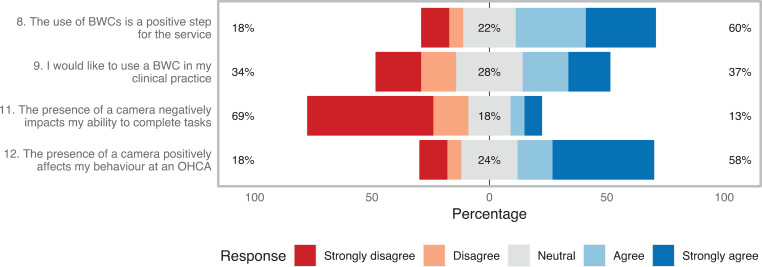
Figure 1. Likert responses to questions 8, 9, 11 and 12.

Note: BWC = body-worn camera; OHCA = out-of-hospital cardiac arrest. There were 18 non-respondents for question 8, and 16 non-respondents for questions 9, 11 and 12.

The respondents were presented with a series of statements regarding the use of BWCs within the context of current pre-hospital service provision. In each case respondents were asked to rate their feelings towards the statement on a Likert scale (see Figure 1). From 65/83 (78%) respondents, 82% gave a positive or neutral response in relation to the use of BWCs in the service. Free text responses included:

- ‘I can see the importance of cameras to gain information.’
- ‘They [BWCs] will continue to be an asset and generally accepted as long as their use is clear and transparent.’
- ‘I think the camera use is a good idea, providing it is used for constructive criticism and not used as a blame tool.’

The most common theme from free text responses associated with a negative response to the use of BWCs was around patient confidentiality; some of these were highly emotive.

- ‘Intrusive and a breach of human rights of the attending crews, patients and patients’ families.’
- ‘I would not know how to answer any questions regarding the use of cameras from patients or other members of the public.’
- ‘We have to be careful that this traumatic event in people’s lives is not turned into some second-rate theatre production.’
- ‘I think if the public knew there [sic] relatives are being filmed they would be aghast.’
- ‘Remove them as soon as possible.’

Subgroup analysis shows that respondents in favour of the use of BWCs were evenly split between paramedics (n = 18, 47%) and paramedic technicians (n = 20, 53%). Of the respondents who felt that BWCs were a service enhancement, 58% (n = 22) were aged between 18 and 35, while all (n = 12, 100%) of those who disagreed with this statement were aged over 35 years old and had worked for more than five years within the ambulance service.

However, the statement ‘I would like to use a BWC in my clinical practice’ did not produce a majority opinion. Free text response analysis indicates that while respondents felt that the use of BWCs in the setting of OHCA analysis and training is of benefit, they were more ambivalent about the prospect of having their own clinical practice audited in this way (Figure 1).

### Impact on practice of body-worn cameras

Lastly, respondents were asked questions regarding the impact of a BWC on clinical practice during OHCA resuscitation. Figure 1 shows that the majority felt that BWCs did not negatively impact their ability to complete tasks, and 58% reported that the presence of a BWC had positively affected their behaviour at an OHCA, or theoretically might do so.

Free text comments on the perceived impact of BWCs on clinical practice reflected the positive, neutral and negative responses:

- ‘It encourages me to examine my personal performance more closely.’
- ‘I usually forget there is a camera present and work as I normally would.’
- ‘The awareness of being filmed makes me self-conscious and hesitant.’

## Discussion

This study represents the first of its kind in the UK, summarising the attitudes of Scottish paramedics to a novel system of BWCs, and video-based audit of pre-hospital resuscitation. The online platform for questionnaire distribution provided an acceptable response rate, which compares well with other surveys of this type (Chesters, Grieve, & Hodgetts, 2016). Additionally, there was no clear bias towards any particular age group of respondents despite the use of an internet-based survey.

The majority of respondents, working with the geographical boundaries of the video audit system, thought that BWCs did not negatively interfere with their ability to do their job. Asking paramedics whether they felt that the use of BWCs was a positive step for the service was felt to be a suitable and appropriate proxy for acceptability, and the majority of respondents felt that the use of a video audit system of this kind was a positive service development. Subgroup demographic analysis highlights those aged over 35 years – generally more experienced paramedics and paramedic technicians – as a group who are particularly cautious about the use of BWCs. These data allow for a more targeted approach to information sharing at the point of implementing any further service developments around the use of video for audit.

Although a small group of respondents reported asking for a camera to be switched off, free text data at the end of the survey, alongside anecdotal evidence, suggest that all these instances occurred within the first few weeks of the cameras’ use, and no further instances have been reported to the video audit team since this survey was performed. It is of critical importance that paramedics do not feel overly uncomfortable in the presence of BWCs to the extent that the quality of care they deliver is inhibited. They must feel able to ask for the BWC to be turned off if they have concerns. These instances, while uncommon, underline the importance of the careful education and training of both viewers and ‘participants’ around the sensitive handling of collected footage.

### Staff concerns

A small number of respondents expressed concerns regarding the use of BWCs in the setting of OHCAs. This is important to note, as a vocal minority could easily derail this type of project. It is possible that these views are under reported, as paramedics who feel negatively towards the project might not engage with attempts to collect feedback.

Taking a broad overview of the free-text comments given by respondents identified the following thematic clusters of concerns:

1. *‘Is recording video of staff and patients without consent ethical or legal?’* While there is no legal barrier to using video for clinical audit or quality improvement, it is still far from the cultural ‘norm’. The research group consulted widely before embarking on the project, seeking guidance from the Central Legal Office, Caldicott Guardian, Director of Public Health, local Data Protection Officer and a Staff and Partnership consultation. Other studies have acknowledged similar challenges in addressing such issues raised by the use of BWCs (Ho et al., 2017), and to date there remains no clear consensus.
2. *‘What about patient confidentiality?’* The authors acknowledge that conducting this project is a delicate balance of perceived intrusion versus the desire for improvement in patient care. Ensuring a robust means of data security remains the project’s highest priority. The research group has developed a bespoke system of data capture, storage and management which includes encryption, system use tracking and an auto-deletion policy. Physical and electronic access to the video footage and the audit tools and hardware remains tightly controlled.
3. *‘Why are we recording video footage at all?’* This highlights a need for clear signposting to the project’s purposes of clinical audit and quality improvement. Footage is not part of the patient’s health records, nor is it to be used for teaching or in training materials. The video is never to be used for assessment of individual performance, though the project’s standard operating procedures do make provision for taking action in the case of observed illegal or gross professional misconduct.
4. *‘Will the video be used against me?’* This has not been explicitly stated by any of the respondents, but appears to underlie a degree of reticence from a small number of staff and their perceived unwillingness to engage with the project and aspects of this survey (e.g. the 20% who chose not to respond to statements suggesting the introduction of BWCs was a positive step for the service). This is first and foremost an issue of organisational trust and is probably the most important aspect in how comfortable front line staff feel with the use of BWCs in a clinical environment. In the context of this project, the 3RU team and Resuscitation Research Group had a good, longstanding relationship with local paramedics which engendered trust, allowing the project to proceed.

### Limitations

The use of a web-based electronic survey did not weigh responses towards any particular demographic group. However, it is possible that the responses were skewed towards those who were more positive about the camera audit, with those holding very negative opinions less likely to contribute to the survey. Those who were critical of the system, however, share highly charged criticism and opposition to the use of BWCs. Further surveys of staff working in a variety of in-hospital and pre-hospital clinical settings would help to provide a deeper and more detailed insight into the acceptability of video-based audit and the attitudes towards BWCs.

## Conclusion

In this cross-sectional survey of paramedics and ambulance technicians in south-east Scotland, the use of BWCs for auditing team performance during an OHCA was seen as acceptable. However, there is a need to ensure ongoing reassurance to crews who are wary of the technology, particularly with respect to patient and staff confidentiality. In 3RU, this has been achieved with sensitive implementation of the system and transparent governance.

## Author contributions

Survey design and results analysis by AD and DL, questions reviewed by DM and GC, survey distributed by DM.

## Conflict of interest

None declared.

## Ethics

Ethical approval for the study was obtained from the Research & Development committees of NHS Lothian and the University of Edinburgh.

## Funding

The camera audit system was funded by a project grant from Chest Heart & Stroke Scotland. AD was part-funded by the Medical Education Directorate, NHS Lothian.
